# High‐efficient and precise base editing of C•G to T•A in the allotetraploid cotton (*Gossypium hirsutum*) genome using a modified CRISPR/Cas9 system

**DOI:** 10.1111/pbi.13168

**Published:** 2019-06-07

**Authors:** Lei Qin, Jianying Li, Qiongqiong Wang, Zhongping Xu, Lin Sun, Muna Alariqi, Hakim Manghwar, Guanyin Wang, Bo Li, Xiao Ding, Hangping Rui, Huimei Huang, Tianliang Lu, Keith Lindsey, Henry Daniell, Xianlong Zhang, Shuangxia Jin

**Affiliations:** ^1^ National Key Laboratory of Crop Genetic Improvement Huazhong Agricultural University Wuhan Hubei China; ^2^ Department of Biosciences Durham University Durham UK; ^3^ Department of Biochemistry School of Dental Medicine University of Pennsylvania Philadelphia PA USA

**Keywords:** cotton (*Gossypium hirsutum*), CRISPR/nCas9 (Cas9 nickase), cytidine deaminase (rAPOBEC1), base editing, off‐target

## Abstract

The base‐editing technique using CRISPR/nCas9 (Cas9 nickase) or dCas9 (deactivated Cas9) fused with cytidine deaminase is a powerful tool to create point mutations. In this study, a novel *G. hirsutum*‐Base Editor 3 (GhBE3) base‐editing system has been developed to create single‐base mutations in the allotetraploid genome of cotton (*Gossypium hirsutum*). A cytidine deaminase sequence (APOBEC) fused with nCas9 and uracil glycosylase inhibitor (UGI) was inserted into our CRISPR/Cas9 plasmid (pRGEB32‐GhU6.7). Three target sites were chosen for two target genes, *GhCLA* and *GhPEBP*, to test the efficiency and accuracy of GhBE3. The editing efficiency ranged from 26.67 to 57.78% at the three target sites. Targeted deep sequencing revealed that the C→T substitution efficiency within an ‘editing window’, approximately six‐nucleotide windows of −17 to −12 bp from the PAM sequence, was up to 18.63% of the total sequences. The 27 most likely off‐target sites predicted by CRISPR‐P and Cas‐OFFinder tools were analysed by targeted deep sequencing, and it was found that rare C→T substitutions (average < 0.1%) were detected in the editing windows of these sites. Furthermore, whole‐genome sequencing analyses on two *GhCLA*‐edited and one wild‐type plants with about 100× depth showed that no *bona fide* off‐target mutations were detectable from 1500 predicted potential off‐target sites across the genome. In addition, the edited bases were inherited to T1 progeny. These results demonstrate that GhBE3 has high specificity and accuracy for the generation of targeted point mutations in allotetraploid cotton.

## Introduction

Single‐nucleotide mutation is the basis of much genetic variation underpinning important agronomic traits in crop plants. Such mutations can result in amino acid substitutions or translation stop codons, which can change the function of the proteins. Therefore, it is desirable to produce novel alleles and improved traits by creating targeted point mutations. Traditional mutagenesis techniques are not targeted and require genome‐scale screening, which is time‐consuming, labour‐intensive and may produce a limited number and type of point mutations. Homologous recombination (HR)‐mediated DNA repair of CRISPR/Cas9 is inefficient for genome editing in plants (Li *et al*., 2013; Mao *et al*., 2013), and the delivery of DNA repair templates is also challenging, hindering the process of precise genome editing. Therefore, it is necessary to develop an alternative genome editing technology that enables genome‐wide and target‐specific editing.

Base‐editing technology (‘Base editor’) is an emerging gene‐targeting modification technology based on the CRISPR system. Base editor is a simple, broadly applicable and efficient technique developed initially by Komor *et al*. (2016). It does not require the generation of DNA double‐strand breaks (DSBs) or DNA templates for efficiently replacing specific bases along the genome. This single‐base‐editing technique can be applied to achieve specific amino acid changes by precisely changing a single base or create a gene knockout by introducing a premature stop codon. The current base‐editing system contains three major components: cytidine deaminase, Cas9 nickase (nCas9) or deactivated Cas9 (dCas9) and uracil glycosylase inhibitor (UGI). The nCas9 and dCas9 are generated by inactivating the enzymatic activity of RuvC or both the RuvC and HNH domains in Cas9 nuclease, which are responsible for cutting two strands of DNA. The nCas9 retains the ability to be programmed with sgRNA and then targets specific DNA sequences to nick one strand at single‐stranded (ssDNA) regions. Subsequently, the cytidine (C) of a ssDNA is converted to uracil (U) via cytidine deaminase, and then, the converted uracil (U) is replaced with thymine (T) during DNA repair or replication process. Serial cytidine base editors, BE1, BE2 and BE3, in mammalian cells were subsequently developed (Komor *et al*., 2016). Several studies then reported successful applications of base‐editing systems in plant species including rice, wheat, tomato and maize (Li *et al*., 2017b; Lu and Zhu, 2017; Ren *et al*., 2018; Shimatani *et al*., 2017; Zong *et al*., 2017). Currently, the most commonly used base‐editing system is the third‐generation base editor, BE3, which is armed with UGI, which inhibits endogenous base excision activity. The resulting base editor converts a cytidine on the nontarget strand to a thymine and cooperates with the mismatch repair system to complete a C•G conversion to a T•A (Standage‐Beier *et al*., 2015). Most recently, adenine base editors (ABEs) have been developed that can convert A•T to G•C in mammalian cells (Gaudelli *et al*., 2017), and this novel system has also been applied in several plant species (Hua *et al*., 2018; Kang *et al*., 2018; Li *et al*., 2018a; Yan *et al*., 2018).

Cotton is an important cash crop for its oilseeds and fibres, valuable for the food and textile industries. *Gossypium hirsutum* (Upland cotton) is a widely cultivated allotetraploid species (A_t_D_t_) with a genome size of 2.5 Gb. Recent progress in cotton genome sequencing has dramatically promoted functional genomics research in this species (Li *et al*., 2015; Wang *et al*., 2018; Yuan *et al*., 2015; Zhang *et al*., 2015). Functional genomics research in cotton still falls some way behind model plant species, but recently, CRISPR/Cas9 and CRISPR/Cpf1 systems have begun to be applied to cotton genome editing (Chen *et al*., 2017; Li *et al*., 2017a, 2019a,b,c; Wang *et al*., 2018). As mentioned above, traditional CRISPR‐Cas9 generates DSBs that trigger complex self‐repairing mechanisms that include nonhomologous end joining (NHEJ) or HR repair. As an allotetraploid species, many alleles in cotton genome are highly homologous with a few SNPs, and so, the traditional CRISPR/Cas9 system is useless when a specific point mutation (base editing) is needed for the functional analysis of homologous alleles.

In this study, two target genes, *GhCLA* and GhPEBP, were selected for their obvious phenotype following mutation. *GhCLA* is a homologous gene to *AtCLA1*, which is responsible for chloroplast development, and its mutant (*cla1*) has an albino phenotype (Mandel *et al*., 1996). VIGS and CRISPR/Cas9 editing of *GhCLA* can generate an albino phenotype in young cotton leaves that is similar to the *cla1* mutant (Gao *et al*., 2013; Li *et al*., 2016; Wang *et al*., 2018). *GhPEBP* participates in the multiplex‐branch developmental process (Chen *et al*., 2018), with a significant phenotype and important agronomic value. We developed an efficient and precise base editor system (GhBE3) consisting of a cytidine deaminase domain fused with nCas9 and UGI, for use in allotetraploid cotton, and which exhibits a high base‐editing efficiency (up to 57.78%, which is comparable with the efficiency in rice and Arabidopsis) with no detectable off‐targets effects.

## Experimental procedures

### Vector construction

**Figure 1:**
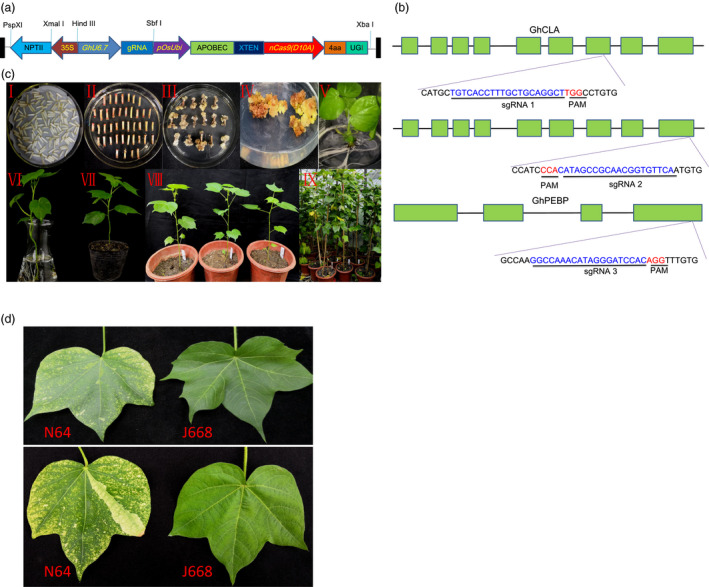
Vector, sgRNA target sites and genetic transformation with GhBE3 system in cotton. (a) Schematic representation of the T‐DNA region of GhBE3 vector. (b) Schematic view of sgRNA1, sgRNA2 and sgRNA3 target sites in the *GhCLA* and *GhPEBP* gene. The target sequences are highlighted in blue, and the PAM sites are highlighted in red. (c) *Agrobacterium*‐mediated genetic transformation and plant regeneration of transgenic plants. (I) Co‐culture stage. (II‐III) Callus induction and differentiation. (IV) Somatic embryogenesis. (V) Plant regeneration. (VI) The acclimatization of regenerated plant in nutrient solution. (VII‐IX) Transgenic plants grown in the greenhouse. (d) Chimeric albino leaves of T0 transgenic N64 plant and green leaves from Jin668 (WT).

In this study, the vector was modified from the vector pRGEB32‐GhU6.7 previously used for cotton genome editing in our laboratory (Wang *et al*., 2018) and contains a neomycin phosphotransferase (NPTII) selection marker; sgRNA transcription was driven by a cotton U6 promoter (GhU6‐7) with very high transcription efficiency. The plasmid pRGEB32‐GhU6.7 was digested by *BstbI* and *XbaI* to delete the Cas9 and replaced by the base editor unit. We amplified cytidine deaminase (APOBEC), Cas9 nickase (nCas9) and uracil glycosylase inhibitor (UGI) units from template plasmid pnCas9‐PBE (Zong *et al*., 2017) and the PCR product was inserted into the binary vector pRGEB32‐GhU6.7 using an In‐Fusion Cloning Kit, thus generating *G. hirsutum*‐Base Editor 3 (GhBE3) (Figure 1a and Appendix S1).

Since the *nCas9* (D10A) gene has a *BsaI* restriction site, the sgRNA expression cassettes could not be introduced using this restriction site. Therefore, the GhBE3 plasmid was linearized with *HpaI* and *SbfI* double digestion, resulting in the deletion of the sgRNA‐terminator fragment. The protocol for sgRNA construction is modified from a previous protocol used for pRGEB32‐GhU6.7 (Wang *et al*., 2018). Two targets of *GhCLA* were designed to be integrated in a single vector, and the tRNA‐sgRNA unit with *HpaI* and *SbfI* double digestions was ligated to the same enzyme digested GhBE3 vector.

### *Agrobacterium*‐mediated cotton transformation

The base‐editing vector was introduced into *Agrobacterium* strain GV3101 by electroporation. Elite cotton (*Gossypium hirsutum*) cultivar Jin668 (Li *et al*., 2019b) was used as the transformation receptor. Seeds were sterilized and cultured in a chamber without light for 6 days at 30 °C. Hypocotyls were cut into 5–10‐mm segments and used as explants for *Agrobacterium‐*mediated transformation following our previous methods (Sun *et al*., 2018; Wang *et al*., 2018).

### On‐target mutation analysis by Sanger sequencing

Genomic DNA was extracted from T_0_ transgenic and WT cotton plants using a DNAquick Plant System (TIANGEN Biotech, Beijing, China). Specific primers (nCas9 F/R and sgRNA F/R in Table S7) for nCas9 and sgRNA sequence were used to confirm transgenics. The target site was amplified with specific primers (CLA F/R and PEBP F/R in Table S7), and the amplicons were ligated into pGEMT‐Easy vector with T4 DNA ligase (Promega, Madison, USA). The PCR products obtained were ligated into the pGEMT‐Easy vector with T4 DNA ligase, the product was transformed into an *E. coli* strain using Top10, and positive clones were used for DNA Sanger sequencing.

### On‐target mutation analysis by targeted deep sequencing

For transgenic plants, a pair of 6 base combination was designed as the barcode tag for each single plant/sample. Each pair of markers was separately added to the 5′ end of the forward and reverse primers for amplifying the target sequence. In total, 14 and 13 barcodes marker were designed for *GhCLA* and *GhPEBP*, respectively (barcode primers of on‐target in Table S5). The corresponding barcode primers were used for PCR amplification of independent samples, and the resulting PCR products were mixed in equal amounts and purified using a purification kit (OMEGA, D2500‐02). One DNA library was applied to Illumina HiSeq 3000 System for paired‐end 150 bp reads.

Raw data from high‐throughput sequencing were analysed by using Trimmomatic software (version 0.32, MINLEN:75) (Bolger *et al*., 2014) to remove low‐quality reads. According to specific barcode primers designed for a single strain, high‐throughput data of target point mutation detection were sorted into each single strain. The high‐throughput sequencing data of the target site detection were also sorted according to the forward and reverse specific primers of each target site. C→T substitution frequency was calculated using customized Perl script.

### Off‐target mutation analysis by targeted deep sequencing

The flanking 500 bp sequence of most potential off‐targets was extracted by Perl script. The target sequences were amplified with barcoded primers (barcode primers of off‐target in Table S6) from genomic DNA in each plant. The C→T substitution frequency in the editing window was calculated for each GhBE3‐edited plant.

### Detection of off‐target mutations by WGS

The genomic DNA was extracted from fresh young leaves (TIANGEN, Cat.#DP305‐03). To identify potential off‐target sites, the BatMis (Tennakoon *et al*., 2012) and Cas‐OFFinder (Bae *et al*., 2014) algorithms were used to compare the two sgRNA target sites of *GhCLA* against the TM‐1 reference genome. The most off‐targets with high off‐score, with C sites in the editing window, and located protein‐coding regions, were identified according to target scores in human and mammalian cells (Hsu *et al*., 2013). Two base‐edited plants (N17, N64) of *GhCLA* and one WT plant were sequenced with 100 × sequencing depth using the Illumina system (HiSeq X Ten). We analysed base‐edited plant variations and compared with WT plants and negative plants to filter out background variations and somaclonal variations. The potential off‐target site mutations were visualized in WT and nCas9‐edited plants by IGV tools to confirm the GhBE3‐induced mutations. All the mutations were visualized using the IGV tool (Robinson *et al*., 2011).

## Results and discussion

### Detection of transgenes in the T0 plants

To test the efficiency of the GhBE3 system in allotetraploid cotton, the endogenous *GhCLA* and *GhPEBP* genes were chosen as targets for base editing. In human cells, base editing accrues within an efficient deamination window (editing window): cytidines within approximately a five‐nucleotide window of −16 to −12 bp from the PAM sequence (Komor *et al*., 2016). However, in plant species such as rice, wheat and maize, the C→T conversions have been found to be induced at seven target sites, with an editing window spanning the position −17 to −11 bp from the PAM sequence (Zong *et al*., 2017). Based on these observations, we designed two sgRNAs (sgRNA1 and sgRNA2) for *GhCLA* and one sgRNA (sgRNA3) for *GhPEBP* (Figure 1a,b and Table S1). Our previous work reported that the cotton endogenous U6 promoter driving a tRNA‐sgRNA transcription system (Wang *et al*., 2018) can enhance CRISPR/Cas9 genome editing in cotton. Therefore, the sgRNAs targeting these endogenous genes were cloned into tRNA‐sgRNA unit and inserted into the binary vector GhBE3. Several independent regenerated (T0) plants were obtained by *Agrobacterium*‐mediated transformation (Figure 1c). For molecular analysis, a total of 46 and 42 independent T0 plants were selected for *GhCLA* (sgRNAs 1 and 2) and *GhPEBP* (sgRNA3), respectively. From PCR analysis using nCas9‐ and sgRNA‐specific primers, 45 independent plants from sgRNA1 and sgRNA2 and 40 independent plants from sgRNA3 were positive transformants, harbouring nCas9, sgRNA fragments (Figure S1), suggesting our cotton transformation system is very effective.

### Detection of on‐target mutations by Sanger sequencing

**Figure 2:**
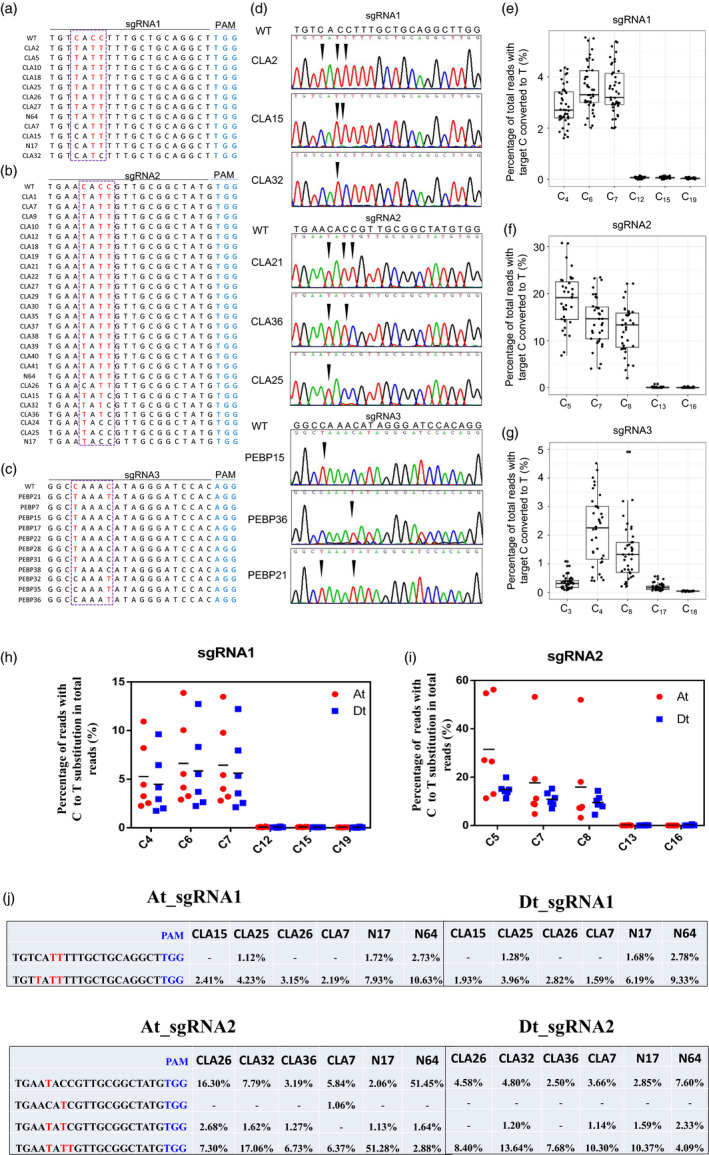
The identification of on‐target mutations by Sanger sequencing and targeted deep sequencing. (a–c) Base‐editing profiles at the sgRNA1, sgRNA2 and sgRNA3 target sites. Each number in the first column (e.g. CLA2) represents an independent GhBE3‐edited plant. (a) 8/12 = triple substitutions; 3/12 = double substitution at C6 and C7; and 1/12 = single substitution at C6. (b) 19/26 = triple substitutions; 4/26 = double substitutions (3 at C5 and C7; 1 at C7 and C8); and 3/26 = single substitution at C5. (c) 1/11 = double substitutions and 10/11 = single substitution (7/11 at C4 and 3/11 at C8). The mutant bases are highlighted in red font, and PAM sequence is highlighted in blue font. (d) Chromatograms of Sanger sequencing indicating the mutated bases at the sgRNA sites of *GhCLA* and *GhPEBP* genes exhibiting different profiles of base substitution. The substitution sites are highlighted by black arrows. (e–g) Base‐editing efficiency of all C sites within sgRNA1, sgRNA2 and sgRNA3 target region revealed by deep sequencing. Substitution efficiency was calculated by the ratio of reads with editing in the total reads at the target region of edited plants. In total 37, 35 and 40 T0 plants were applied for deep sequencing at sgRNA1, sgRNA2 and sgRNA3 target sites, respectively. (h,i) Percentage of reads with target C→T substitution in total reads at sgRNA1 and sgRNA2 at target sites in At (red dot) and Dt subgenomes (blue square) of cotton. (j) The C→T substitution profiles in At and Dt subgenomes of six T0 edited cotton plants at sgRNA1 (upper panel) and sgRNA2 target site (lower panel). The PAM sites were indicated with blue font, and the target C→T substitutions were indicated with red font. ‘‐’ means no detectable (lower than 1%) C→T substitution at the target sites.

In order to test the viability and efficacy of GhBE3 in cotton, 45 independent transgenic T0 plants of *GhCLA* and 40 independent T0 plants of *GhPEBP* were analysed by Sanger sequencing. The sequencing data showed that 12 out of the 45 plants contained at least one C→T substitution at the sgRNA1 target region of *GhCLA* (with editing efficiency of 26.67%) and 26 out of the 45 plants exhibited at least one C→T substitution at the sgRNA2 target region of *GhCLA* (with editing efficiency of 57.78%) (Table 1). For the *GhPEBP* transgenic plants, Sanger sequencing data showed that 11 out of 40 plants contained at least one C→T substitution at the sgRNA3 target region (with editing efficiency of 27.5%) (Table 1). Among these T0 plants with the base editing, we found that there were three or four types of mutations in the editing window (Figure 2a–d). For sgRNA1, only one plant (CLA32) showed the single C→T substitution at position C6, whereas the other 11 plants harboured two or three substitutions (C6, C7 or C4, C6, C7). (Table 2 and Figure 2a). Among these 26 edited plants at the sgRNA2 target, there were only three plants that harboured the single C→T substitution, the remaining mutants occurring simultaneously at two or three sites, of these, 19/26 = triple substitutions; 4/26 = double substitutions (3 at C5 and C7; 1 at C7 and C8) and 3/26 = single substitution at C5 (Table 2 and Figure 2b). Among the 11 edited plants at the target sgRNA3, according to Figure 2c, 10/11 plants were single substitutions (7 at C4 and 3 at C8 positions, respectively) and only one (PEBP21) had double substitutions (Table 2 and Figure 2c).

**Table 1:** Summary of base‐editing efficiency of *GhCLA* and *GhPEBP* genes

Target gene	sgRNA	The number of transgenic plants	The number of plants with base editing	Base‐editing efficiency
*GhCLA*	sgRNA1	45	12	26.67%
*GhCLA*	sgRNA2	45	26	57.78%
*GhPEBP*	sgRNA3	40	11	27.5%

**Table 2:**
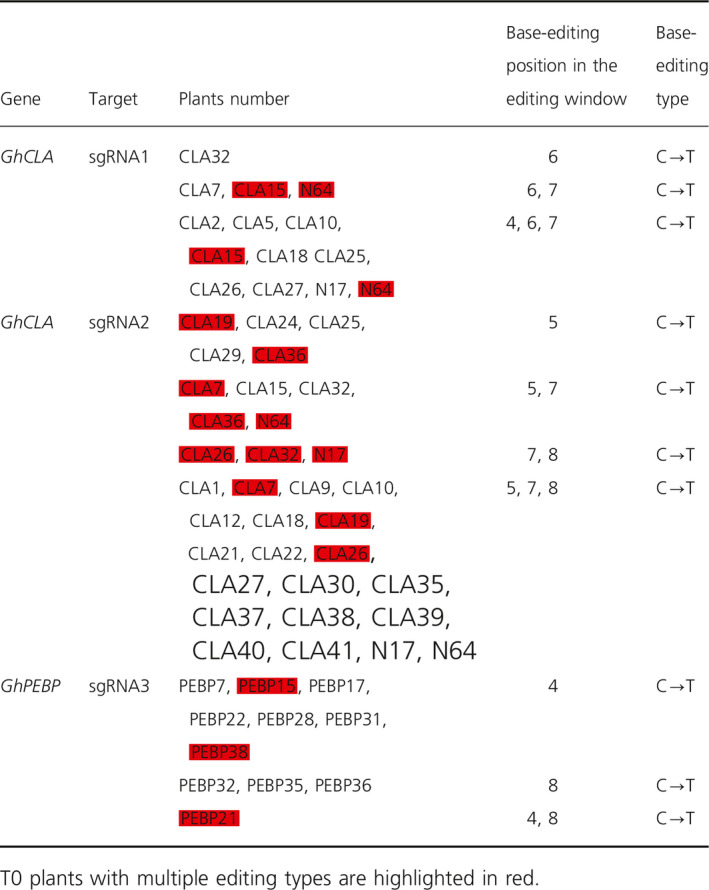
Details of base‐editing profile at three sgRNA target sites

The above data show that GhBE3 exhibits efficient base editing in cotton. The base substitution was detected within the editing window from C_3_ to C_8_ of the protospacer, which is consistent with editing windows reported in animals and other crops (Komor *et al*., 2016; Zong *et al*., 2017). We found that the C→T substitution efficiency reached up to 57.78%, which is comparable with the efficiency in rice, maize and wheat (Zong *et al*., 2017). Most common mutation types in the editing window of the *GhCLA* target were multiple substitutions, which are also consistent with results reported in other crops (Li *et al*., 2017b; Zong *et al*., 2017). However, the sgRNA3 target of this study showed a higher probability of single point mutation, which may be related to the particular sequence of the sgRNA. Although the editing efficiency was higher in *GhCLA*, the predicted phenotype (albino seedlings) of the transgenic plants did not show up in most T0 plants, and only one plant exhibited a chimeric phenotype (Figure 1d). The absence of the phenotype in the edited plants might be due to the possibility that the point mutation (base substitution) in the target site would only change a single amino acid (or not, if base editing occurred at the ‘wobble’, the 3^rd^ base of a codon) and no frameshift happened. In this case, the resultant proteins will likely have full function, and so, no obvious phenotype would be detected. For the chimeric phenotype, we detected by deep sequencing about 8% indels (most are deletions) and speculate that the chimeric phenotype may be caused by frameshift mutation rather than base substitution (Figure S2).

### High‐throughput deep sequencing for on‐target base editing in *GhCLA‐* and *GhPEBP‐*edited plants

Sanger sequencing for target sequence analysis is very reliable, but the rate of throughput of this method is very limited, and the cost is relatively high. Deep sequencing on the other hand is very reliable for the detection of various kinds of mutations, with high throughput and low cost. Barcode‐based high‐throughput sequencing has been applied for genotyping of a range of target genes in zebrafish and CRISPR/Cas9‐edited plant species (Varshney *et al*., 2016; Wang *et al*., 2018). In this report, we designed a pair of specific barcodes to mark each independent transgenic plant and attached them to a pair of forward and reverse site‐specific primers corresponding to the targeted genes, for PCR (Table S5). As shown in Figure 2e–g and Table S2, the C→T substitution ratio within editing window of all sgRNAs ranged from 0.34% to 18.63% of the total DNA sequences. For the sgRNA2 site, 18.63%, 14.20% and 12.44% C→T substitutions at C_5_, C_7_ and C_8_ were detected from the 26 000 reads, and 2.87%, 3.58% and 3.47% of C→T substitutions at C_4_, C_6_ and C_7_ at sgRNA1 sites were identified from 440 000 reads. For the sgRNA3 site, 0.34%, 2.77% and 1.51% of C→T substitutions at C_3_, C_4_ and C_8_ were observed from 101 000 reads. A higher base‐editing ratio of *GhCLA* was observed in the A‐subgenome than in the D‐subgenome (4.28% vs 1.75% at sgRNA1, 20.30% vs 15.89% at sgRNA2). Obviously, the efficiency of C→T substitutions in the editing window is significantly higher than other C sites in the sgRNA sequence (20 nt in length), which illustrates how the GhBE3 performed effective base editing in the target editing window of sgRNA sites. Since Gossypium hirsutum (Upland cotton) is a heterotetraploid species with At and Dt subgenomes, we analyzed the genotypes of the sgRNA1 and sgRNA2 target sites in the At and Dt subgenomes of six T0 cotton plants. The data show that there has no obvious bias of base editing between At and Dt subgenomes (Figure 2h), with the editing efficiency ranging from 1.12 to 10.63% in At subgenome at sgRNA1 target site, while 1.28 to 9.33% in Dt subgenome at sgRNA1 target site (Figure 2j). At the sgRNA2 target site, the editing efficiency ranged from 1.13 to 51.45% in At subgenome, while 1.14 to 10.37% in Dt subgenome (Figure 2j).

The results from the barcode strategy and from Sanger sequencing were identical in both *GhCLA‐* and *GhPEBP*‐edited T0 plants. We found that base‐editing efficiency at the sgRNA2 site was much higher than at either the sgRNA1 or the sgRNA3 site, using either Sanger sequencing or barcode‐based high‐throughput sequencing data. In accordance with the previous results, the difference in editing efficiency at the three sgRNAs might be due to the limitations of suitable sgRNA target sites, nucleotide composition, GC content or sgRNA secondary structure (Liang *et al*., 2016; Ma *et al*., 2015), and this requires further investigation.

### Target deep sequencing reveals no off‐target effect at most potential off‐target sites

**Figure 3:**
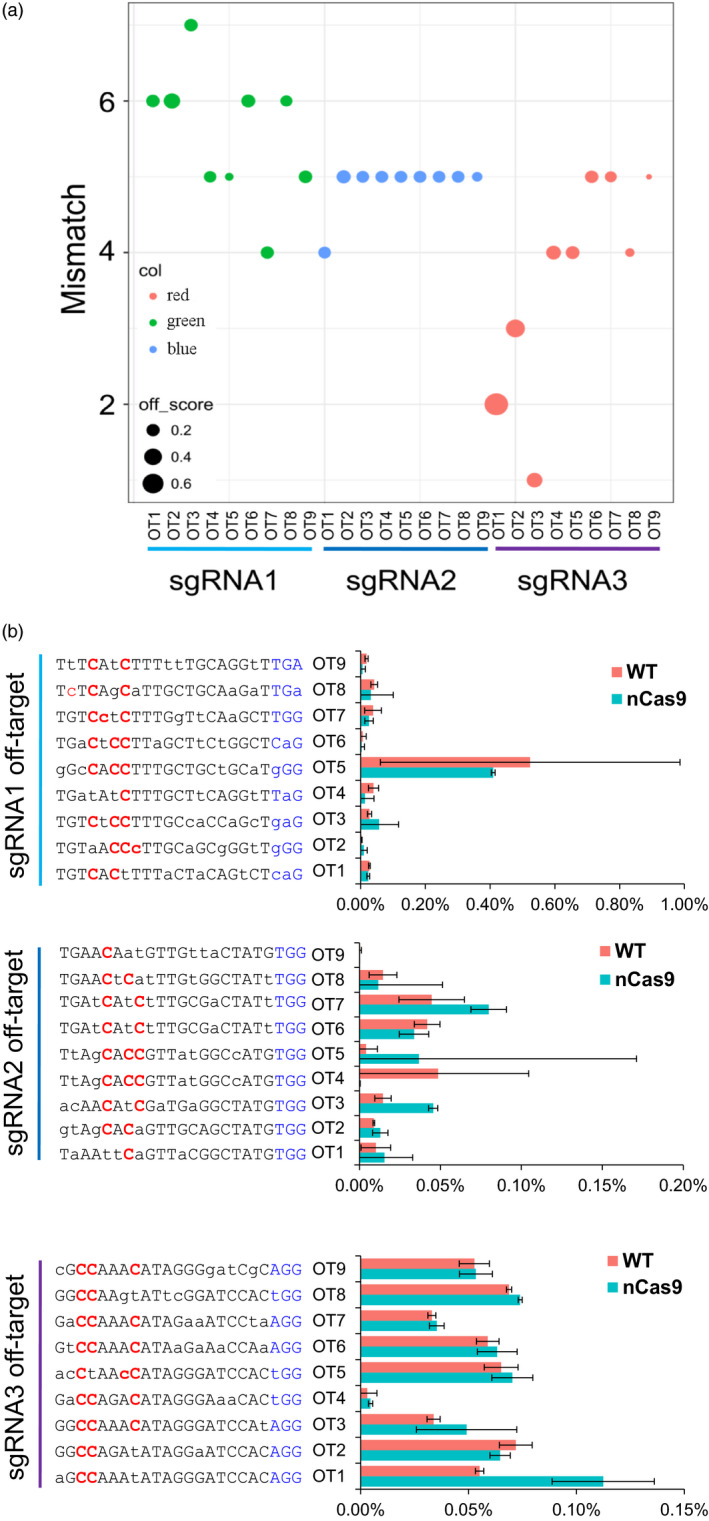
The evaluation of off‐target effect of GhBE3 in edited cotton plants by targeted deep sequencing. (a) Scores (high off‐target score and the seed sequences ≤ 5 mismatches with the sgRNA sequences) for the most potential off‐target sites. The number of mismatches shown in the figure is from both sgRNA and PAM sequences (23 nt in length). (b) No off‐target mutation was detected at most potential off‐target sites in sgRNA1, sgRNA2 and sgRNA3 by targeted deep sequencing. There were 12, 26 and 10 edited plants in sgRNA1, sgRNA2 and sgRNA3 used to detect off‐target and 3 WT plants used for analysis in this experiment. Error bar = 3. Mismatch bases of sgRNA between the WT and off‐target sequences are marked with lowercase letters, and all C bases in editing window of off‐target are highlighted in red font.

The above data reveal that GhBE3 exhibits considerable editing efficiency. To detect any potential off‐target effects of this system, we computationally predicted all off‐target sites for the three sgRNAs and mapped their seed sequences to the TM‐1 reference genome using CRISPR‐P and Cas‐OFFinder tools (seed sequences ≤ 5 mismatches with the sgRNA sequences) (Bae *et al*., 2014; Liu *et al*., 2017). We detected 1001, 499 and 1180 potential off‐target sites for sgRNA1, sgRNA2 and sgRNA3, respectively (Table S4). Nine most likely off‐target sites were selected for each sgRNA with a high off‐score, and most sites were located in protein‐coding regions (Figure 3a and Table S8). The base‐editing frequency was analysed at these nine off‐target sites for 26 edited plants using sgRNA1, 12 edited plants using sgRNA2 and 10 edited plants using sgRNA3 by deep sequencing. WT plants were also included as controls for deep sequencing and the subsequent analysis. The data indicate that the C→T substitution ratio in the editing window of the most likely off‐target sites was lower than 0.1% (Figure 3b and Table S3). No difference in the off‐target ratio between base‐edited and WT plants was observed after statistical analysis.

These results reveal that the GhBE3 has a low off‐target efficiency in cotton. It has been reported that base‐editing systems have varying levels of off‐target effects in different species. Kim *et al*. (2017) used digested‐genome sequencing (Digenome‐seq) to assess the specificity of base editor in the human genome and detected a substitution frequency of 0.1% at the off‐target sites. In another report, deep sequencing analysis detected approximately 0.14–0.38% off‐target mutations in a base‐edited tomato genome (Shimatani *et al*., 2017). Comparing the off‐target effects in previous reports, the GhBE3 system used in this report is highly precise for cotton genome editing. The negligible C→T conversions at these off‐target sites in this report may be due to sequencing errors or inherent genetic and/or somaclonal variations during the tissue culture process, which is consistent with our recent report regarding the off‐target evaluation of CRISPR/Cas9‐edited cotton plants (Li *et al*., 2019a).

### Whole‐genome sequencing analysis of off‐target effects of GhBE3 in cotton

**Figure 4:**
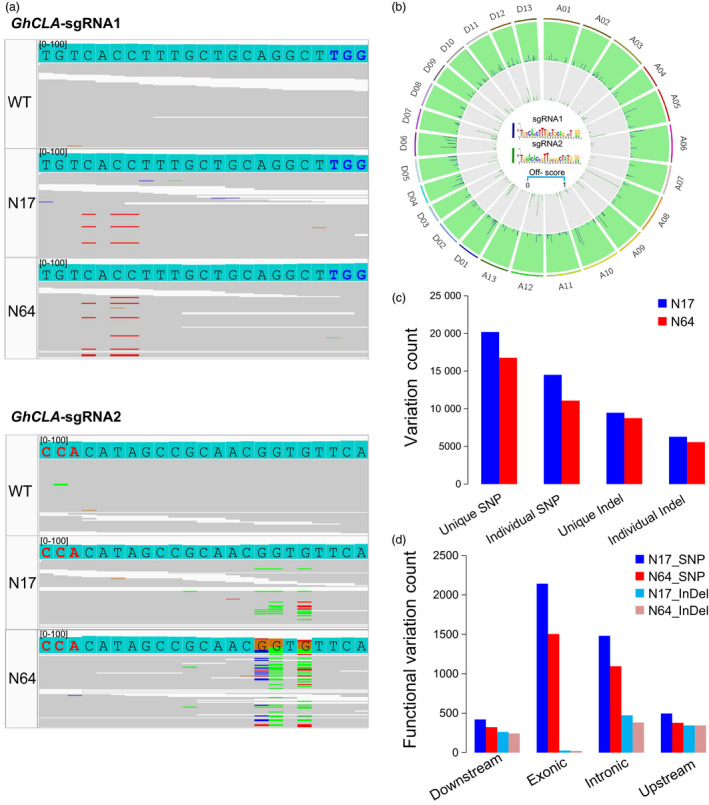
Genome‐wide analysis of off‐target effect for the GhBE3 system in cotton plants by whole‐genome sequencing. (a) Sequence alignments at the sgRNA1 and sgRNA2 target sites by IGV. Low C→T mutations detected at the C_8_ site of sgRNA2 target region. PAM sequence of sgRNA1 and sgRNA2 is highlighted in blue and red, respectively. Red lines represent thymine (T), blue lines represent cytidine (C), and green lines represent adenine (A) at the sequencing data. (b) Genome‐wide Circos plot represents off‐target site scores for sgRNA1 (blue) and sgRNA2 (green). The off‐target sequences are highlighted in the Circos centre. (c,d) Annotation of SNPs and indels in the downstream (transcription start site 2k), exonic, intronic, upstream (transcription end site 2k) and intergenic regions of N17 and N64 GhBE3‐edited T0 plants genome. The unique variation presents in N17 and N64 plants, but not in WT and negative control plants. The individual variation represents mutations except the overlap variations in N17 and N64 plants.

To assess GhBE3 off‐target effects in cotton plants on a genome‐wide scale, whole‐genome sequencing (WGS) was performed for two base‐edited plants (N17, N64) for the *GhCLA* gene and one WT plant, with 100 × sequencing depth. Furthermore, two WT plants and three T0 negative (Ne) plants (following tissue culture and plant generation but without T‐DNA insertion, that is no CRISPR/Cas9 component) identified in our recent reports were also included in this analysis since they share a very similar genetic background with the WT and GhBE3‐edited plants (Li *et al*., 2019a). We identified all potential off‐targets of these three sgRNAs at a whole‐genome scale through bioinformatics analysis. In total, 499 off‐targets for sgRNA1 and 1001 off‐targets for sgRNA2 were identified, based on Cas‐OFFinder analysis (Table S4 and Figure 4c). Firstly, we checked the on‐target base editing at sgRNA1 and sgRNA2 target sites using Integrative Genomics Viewer (IGV), and results showed many C→T substitutions in the editing window of sgRNA1 target regions (Figure 4a), consistent with the Sanger sequencing data shown in Figure 2. Similarly, C→T mutations were also detected at the editing window of the sgRNA2 target region, but the ratio was lower than that in the sgRNA1 target region, although many C→G mutations were detected at this site (Figure 4b). Similar results have been detected in rice (G→C or G→T mutations) (Li *et al*., 2017b), and it is not clear how these kinds of unintended base mutations were generated.

To evaluate potential off‐target mutations of GhBE3 at a whole genome‐wide scale, two variant caller tools with strict parameter settings were applied to get the high concordance variations (Table S9). In total, 976, 280, 11,124,776 and 1,138,453 SNPs, and 134,097, 139,787 and 140,733 indels were detected in WT, N17 and N64 plants, respectively, compared with the TM‐1 reference genome. We used two negative control plants and two WT plants from a previous study to eliminate somaclonal variations or germ‐line background variations (Table 3) (Li *et al*., 2019a). According to the analysis, the GhBE3‐induced mutations (on‐target editing) are solely present in GhBE3‐edited plants, but not in WT and negative control plants. Finally, a total of 20 193 and 16 770 SNPs and 9471 and 8756 indels were detected in N17 and N64 plants, respectively (Figure 4c and Table 3). These variations were annotated by ANNOVAR tools. The 21.5% functional variations were detected where the exonic region contained a high ratio (Figure 4d). 5689 SNPs and 3189 indels were shared between the N17 and N64 plants (Figure 4c). After filtering the shared variations, the remaining individual variations (14 504 and 11 081 SNPs and 6280 and 5565 indels in the N17 and N64 plants, respectively) were filtered for further detection of GhBE3‐induced off‐target mutations (Table 3). These variations overlapped with the 1500 predicted potential off‐target sites by Cas‐OFFinder. No *bona fide* off‐target mutations were detected at these potential off‐target sites. The targeted deep sequencing and WGS data therefore suggest that GhBE3 did not cause detectable off‐target mutations on a whole genome‐wide scale and was highly precise for cotton genome editing.

**Table 3:** Summary of high confidence variations in wild‐type, negative control and GhBE3‐edited cotton plants through whole‐genome sequencing

Description	Plants VS Ref		Plants VS Ref/WT		Plants VS Ref/WT/Ne		Individual variations	
	SNP	indel	SNP	indel	SNP	Indel	SNP	Indel
WT (s195)	976 280	134 097	–	–	–	–	–	–
WT (s79)*	1 211 622	149 327	–	–	–	–	–	–
WT (s199)*	1 210 509	148 567	–	–	–	–	–	–
Negative (s65)*	1 203 206	149 636	319 900	19 300	–	–	–	–
Negative (s66)*	1 217 124	148 842	329 233	18 456	–	–	–	–
Negative (s67)*	1 209 155	135 845	339 335	29 969	–	–	–	–
*nCas9‐CLA (N17)*	1 124 776	139 787	380 873	22 635	20 193	9471	14 504	6280
*nCas9‐CLA (N64)*	1 138 453	140 733	351 187	21 541	16 770	8756	11 081	5565

The ‘Plants vs Ref’ represents the high confidence variations of per plant compared with TM‐1 reference genome (Table S9). The ‘Plants vs Ref/WT’ represents the variations of per plant compared with TM‐1 and wild type (WT). Similarly, the ‘Plants vs Ref/WT/Ne (unique variation)’ represents the variations of per GhBE3‐edited plants compared with TM‐1, WT and negative plants. The individual variations indicated that two base‐edited plants have the similar genotype as three negative plants, but differ from each other and contain specific variations, which may include the GhBE3‐induced off‐target mutations. Sample‐specific variations (including pre‐existing variations, and/or inherent variations and/or nCas9‐induced mutations) were annotated by ANNOVAR (Figure 4c). The ‘*’ indicates the genome data from these samples were cited from our previous report (Li *et al*., 2019a).

### The base editing produced by GhBE3 was genetically inheritable from T0 to T1 progeny

**Figure 5:**
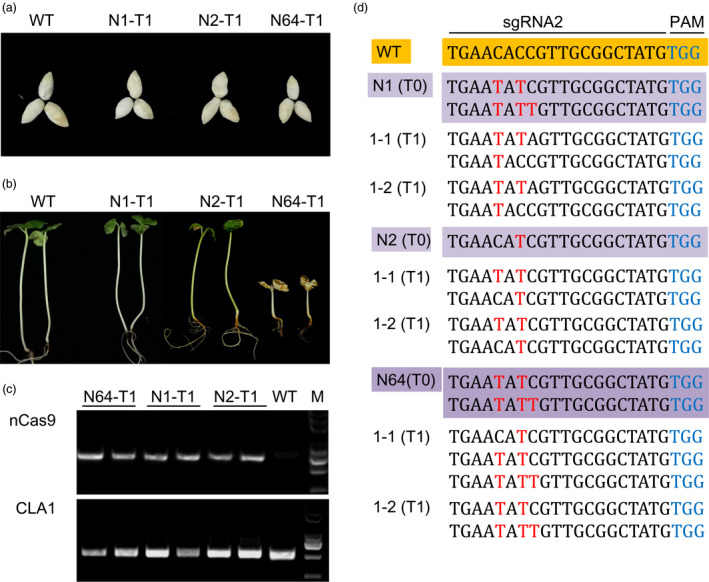
Base editing of *GhCLA* was genetically inheritable to T1 progenies. (a,b) Seeds and their young seedlings from WT, N1, N2 and N64 T0 parental plants. (c) PCR analysis to detect *nCas9* and *GhCLA* genes in WT, N1, N2 and N64 T1 progenies. (d) Genotyping of independent T0 plants and their T1 progeny at the sgRNA2 of *GhCLA* gene. The WT and T0 plants are highlighted in orange and purple background colour, respectively. Base‐editing sites are highlighted in red font, and PAMs are highlighted in blue font.

The above analysis allowed us to evaluate the base‐editing efficiency and accuracy in stable transgenic cotton T0 plants using Sanger sequencing, target deep sequencing and whole‐genome sequencing. In order to test the inheritance of base editing induced by GhBE3 from T0 to T1 progeny, the T1 plants generated from N1, N2 and N64 T0 plants of *GhCLA* were analysed by PCR amplification and Sanger sequencing. T1 seeds and their seedlings were obtained from T0 positive parental seedlings, as shown in Figure 5a,b. The N64 T1 plantlets exhibited a complete albino phenotype as expected (Figure 5b), whereas other T1 plantlets from two lines N1 and N2 remained green. All the T1 plantlets were checked with nCas9‐ and CLA1‐specific primers (Table S7) to identify positive transformants and target amplifications (Figure 5c). The results showed that C→T substitutions at sgRNA targets were detected in T1 generation plants from three T0 parental plants. Furthermore, several newly generated base‐editing types at the base‐editing window of sgRNA2 site were detected from T_1_ progenies of N1, N2 and N64 T0 plantlets (Figure 5d). These results confirmed that the base editing was heritable (Figure 5). In addition to the predicted mutations inherited from T0 plants, several new mutations were detected in T1 plants but not in T0 plants. New mutations generated in the T1 progeny at the target sites indicate that the base editor (GhBE3) can continue to induce base editing in the progeny containing the T‐DNA insertion. This means that, to ensure stability of edited progeny, it will be necessary to screen for offspring without T‐DNA insertions, so that the mutations in the parental generation will be faithfully inherited to the next generation while preventing the occurrence of additional mutations. Recently, a report showed that base editing mediated by the rBE3 system could be stably and faithfully inherited to the T1 generation in rice, and homozygous mutants without exogenous T‐DNA could be obtained in T1 generation (Ren *et al*., 2017).

In conclusion, GhBE3 can efficiently perform C to T substitutions on sgRNA targets in the cotton genome, and successful base editing of two endogenous genes prompted us to apply this system to future functional genomics studies. More importantly, no off‐target C→T substitutions were detected at potential off‐target sites in a genome‐wide scale analysis. Therefore, it is a feasible and effective tool for targeted base editing in cotton and will provide important technical support for the functional analysis of cotton genome, genetic improvement of crops and breeding of new varieties. The combination of adenine and cytidine base editors can now generate all four base transition mutations, and it has now been shown that adenine and cytidine base editing can be achieved simultaneously in rice (Hua *et al*., 2019) and will also be applied in cotton in the near future.

## Author contributions

S.X.J. and X.L.Z. designed the Project. L.Q. performed experiments and wrote the manuscript. J.Y.L., Q.Q.W. and Z.P.X performed genotype data and WGS data analysis. S.X.J., J.Y.L., M.A., H.M and K.L. revised the manuscript. All authors read and approved the final manuscript.

## Conflict of interest

The authors have declared that no competing interests exist in this manuscript.

## Supporting information


**Appendix S1** Sequences of each component of GhBE3.
**Figure S1** PCR analysis for putative transgenic T0 plants with nCas9 and sgRNA specific primers.
**Figure S2** Insertion, deletion and substitutions sizes of sgRNA2 in the *GhCLA*.
**Table S1** Summary of target gene in APOBEC1 base editing.
**Table S5** Barcode primers for detecting on‐target in independent T0 transgenic plants with deep sequencing.
**Table S6** Barcode primers for detecting off‐target in base editing T0 transgenic plants with deep sequencing.
**Table S7** Primers used for vectors construction, positive test and target amplification.
**Table S8** The nine most potential off‐target sites of each target in cotton.
**Table S9** Summary of variation calling between wild‐type (WT) and GhBE3 edited plants by the samtools and GATK.

**Table S2** Editing of on‐target in GhBE3 transgenic plants by deep sequencing.

**Table S3** Summary of deep sequencing results for most potential off‐target site.

**Table S4** Summary of genome‐wide potential off‐targets predictions by CRISPR‐P and Cas‐OFFinder tools.

## Data Availability

The target deep sequencing and WGS data have been submitted to the NCBI Sequence Read Archive (SRA) BioProject ID: PRJNA380842.
