# Supplementary material for: High‐efficient and precise base editing of C•G to T•A in the allotetraploid cotton (Gossypium hirsutum) genome using a modified CRISPR/Cas9 system
Source: Plant Biotechnol J. 2019 Jun 7;18(1):45–56. doi: 10.1111/pbi.13168 (PMC6920158; doi:10.1111/pbi.13168)
Supplement: Supplementary file 1 — Appendix S1 Sequences of each component of GhBE3. Figure S1 PCR analysis for putative transgenic T0 plants with nCas9 and sgRNA specific primers. Figure S2 Insertion, deletion and substitutions sizes of sgRNA2 in the GhCLA. Table S1 Summary of target gene in APOBEC1 base editing. Table S5 Barcode primers for detecting on‐target in independent T0 transgenic plants with deep sequencing. Table S6 Barcode primers for detecting off‐target in base editing T0 transgenic plants with deep sequencing. Table S7 Primers used for vectors construction, positive test and target amplification. Table S8 The nine most potential off‐target sites of each target in cotton. Table S9 Summary of variation calling between wild‐type (WT) and GhBE3 edited plants by the samtools and GATK. [file PBI-18-45-s003.doc]

**Supporting Information**

**Appendix S1.** Sequences of each component of GhBE3.

**Figure S1.** PCR analysis for putative transgenic T0 plants with nCas9 and sgRNA specific primers.

**Figure S2.** Insertion, deletion and substitutions sizes of sgRNA2 in the *GhCLA.*

**Table S1.** Summary of target gene in APOBEC1base editing.

**Table S2.** Summary of on-target editing profile in GhBE3 edited plants by deep sequencing. (sgRNA1)

**Table S3.** Summary of deep sequencing results for most potential off-target site.

**Table S4.** Summary of genome-wide potential off-targets predictions by CRISPR-P and Cas-OFFinder tools.

**Table S5.** Barcode primers for detecting on-target in independent T0 transgenic plants with deep sequencing.

**Table S6.** Barcode primers for detecting off-target in base editing T0 transgenic plants with deep sequencing.

**Table S7.** Primers used for vectors construction, positive test and target amplification.

**Table S8.** The nine most potential off-target sites of each target in cotton.

**Table S9.** Summary of variation calling between wild-type (WT) and GhBE3 edited plants by the samtools and GATK.

**AppendixS1: The sequences of each component of GhBE3 system.** The sequence of APOBEC1 indicated by black font , XTEN (green), nCas9(D10A) (red), UGI (blue) and SV40 NLS (orange) .

ccaaagaagaagaggaaggtttcatcggagaccggccctgttgctgttgaccccaccctgcggcggagaatcgagccacacgagttcgaggtgttcttcgacccaagggagctccgcaaggagacgtgcctcctgtacgagatcaactggggcggcaggcactccatctggaggcacaccagccaaaacaccaacaagcacgtggaggtcaacttcatcgagaagttcaccaccgagaggtacttctgcccaaacacccgctgctccatcacctggttcctgtcctggagcccatgcggcgagtgctccagggccatcaccgagttcctcagccgctacccacacgtcaccctgttcatctacatcgccaggctctaccaccacgccgacccaaggaacaggcagggcctccgcgacctgatctccagcggcgtgaccatccaaatcatgaccgagcaggagtccggctactgctggaggaacttcgtcaactactccccaagcaacgaggcccactggccaaggtacccacacctctgggtgcgcctctacgtgctcgagctgtactgcatcatcctcggcctgccaccatgcctcaacatcctgaggcgcaagcaaccacagctgaccttcttcaccatcgccctccaaagctgccactaccagaggctcccaccacacatcctgtgggctaccggcctcaagtccggcagcgagacgccaggcacctccgagagcgctacgcctgaacttaaggacaagaagtactcgatcggcctcgccatcgggacgaactcagttggctgggccgtgatcaccgacgagtacaaggtgccctctaagaagttcaaggtcctggggaacaccgaccgccattccatcaagaagaacctcatcggcgctctcctgttcgacagcggggagaccgctgaggctacgaggctcaagagaaccgctaggcgccggtacacgagaaggaagaacaggatctgctacctccaagagattttctccaacgagatggccaaggttgacgattcattcttccaccgcctggaggagtctttcctcgtggaggaggataagaagcacgagcggcatcccatcttcggcaacatcgtggacgaggttgcctaccacgagaagtaccctacgatctaccatctgcggaagaagctcgtggactccaccgataaggcggacctcagactgatctacctcgctctggcccacatgatcaagttccgcggccatttcctgatcgagggggatctcaacccagacaacagcgatgttgacaagctgttcatccaactcgtgcagacctacaaccaactcttcgaggagaacccgatcaacgcctctggcgtggacgcgaaggctatcctgtccgcgaggctctcgaagtccaggaggctggagaacctgatcgctcagctcccaggcgagaagaagaacggcctgttcgggaacctcatcgctctcagcctggggctcaccccgaacttcaagtcgaacttcgatctcgctgaggacgccaagctgcaactctccaaggacacctacgacgatgacctcgataacctcctggcccagatcggcgatcaatacgcggacctgttcctcgctgccaagaacctgtcggacgccatcctcctgtcagatatcctccgcgtgaacaccgagatcacgaaggctccactctctgcctccatgatcaagcgctacgacgagcaccatcaggatctgaccctcctgaaggcgctggtccgccaacagctcccggagaagtacaaggagattttcttcgatcagtcgaagaacggctacgctgggtacatcgacggcggggcctcacaagaggagttctacaagttcatcaagccaatcctggagaagatggacggcacggaggagctcctggtgaagctcaacagggaggacctcctgcggaagcagagaaccttcgataacggcagcatcccccaccaaatccatctcggggagctgcacgccatcctgagaaggcaagaggacttctaccctttcctcaaggataaccgggagaagatcgagaagatcctgaccttcagaatcccatactacgtcggccctctcgcgcgggggaactcaagattcgcttggatgacccgcaagtctgaggagaccatcacgccgtggaacttcgaggaggtggtggacaagggcgctagcgctcagtcgttcatcgagaggatgaccaacttcgacaagaacctgcccaacgagaaggtgctccctaagcactcgctcctgtacgagtacttcaccgtctacaacgagctcacgaaggtgaagtacgtcaccgagggcatgcgcaagccagcgttcctgtccggggagcagaagaaggctatcgtggacctcctgttcaagaccaaccggaaggtcacggttaagcaactcaaggaggactacttcaagaagatcgagtgcttcgattcggtcgagatcagcggcgttgaggaccgcttcaacgccagcctcgggacctaccacgatctcctgaagatcatcaaggataaggacttcctggacaacgaggagaacgaggatatcctggaggacatcgtgctgaccctcacgctgttcgaggacagggagatgatcgaggagcgcctgaagacgtacgcccatctcttcgatgacaaggtcatgaagcaactcaagcgccggagatacaccggctgggggaggctgtcccgcaagctcatcaacggcatccgggacaagcagtccgggaagaccatcctcgacttcctcaagagcgatggcttcgccaacaggaacttcatgcaactgatccacgatgacagcctcaccttcaaggaggatatccaaaaggctcaagtgagcggccagggggactcgctgcacgagcatatcgcgaacctcgctggctcccccgcgatcaagaagggcatcctccagaccgtgaaggttgtggacgagctcgtgaaggtcatgggccggcacaagcctgagaacatcgtcatcgagatggccagagagaaccaaaccacgcagaaggggcaaaagaactctagggagcgcatgaagcgcatcgaggagggcatcaaggagctggggtcccaaatcctcaaggagcacccagtggagaacacccaactgcagaacgagaagctctacctgtactacctccagaacggcagggatatgtacgtggaccaagagctggatatcaaccgcctcagcgattacgacgtcgatcatatcgttccccagtctttcctgaaggatgactccatcgacaacaaggtcctcaccaggtcggacaagaaccgcggcaagtcagataacgttccatctgaggaggtcgttaagaagatgaagaactactggaggcagctcctgaacgccaagctgatcacgcaaaggaagttcgacaacctcaccaaggctgagagaggcgggctctcagagctggacaaggccggcttcatcaagcggcagctggtcgagaccagacaaatcacgaagcacgttgcgcaaatcctcgactctcggatgaacacgaagtacgatgagaacgacaagctgatcagggaggttaaggtgatcaccctgaagtctaagctcgtctccgacttcaggaaggatttccagttctacaaggttcgcgagatcaacaactaccaccatgcccatgacgcttacctcaacgctgtggtcggcaccgctctgatcaagaagtacccaaagctggagtccgagttcgtgtacggggactacaaggtttacgatgtgcgcaagatgatcgccaagtcggagcaagagatcggcaaggctaccgccaagtacttcttctactcaaacatcatgaacttcttcaagaccgagatcacgctggccaacggcgagatccggaagagaccgctcatcgagaccaacggcgagacgggggagatcgtgtgggacaagggcagggatttcgcgaccgtccgcaaggttctctccatgccccaggtgaacatcgtcaagaagaccgaggtccaaacgggcgggttctcaaaggagtctatcctgcctaagcggaacagcgacaagctcatcgccagaaagaaggactgggacccaaagaagtacggcgggttcgacagccctaccgtggcctactcggtcctggttgtggcgaaggttgagaagggcaagtccaagaagctcaagagcgtgaaggagctcctggggatcaccatcatggagaggtccagcttcgagaagaacccaatcgacttcctggaggccaagggctacaaggaggtgaagaaggacctgatcatcaagctcccgaagtactctctcttcgagctggagaacggcaggaagagaatgctggcttccgctggcgagctccagaaggggaacgagctcgcgctgccaagcaagtacgtgaacttcctctacctggcttcccactacgagaagctcaagggcagcccggaggacaacgagcaaaagcagctgttcgtcgagcagcacaagcattacctcgacgagatcatcgagcaaatctccgagttcagcaagcgcgtgatcctcgccgacgcgaacctggataaggtcctctccgcctacaacaagcaccgggacaagcccatcagagagcaagcggagaacatcatccatctcttcaccctgacgaacctcggcgctcctgctgctttcaagtacttcgacaccacgatcgatcggaagagatacacctccacgaaggaggtcctggacgcgaccctcatccaccagtcgatcaccggcctgtacgagacgaggatcgacctctcacaactcggcggggataagagacccgcagcaaccaagaaggcagggcaagcaaagaagaagaagacgcgtgactccggcggcagcaccaacctgtccgacatcatcgagaaggagacgggcaagcaactcgtgatccaggagagcatcctcatgctgccagaggaggtggaggaggtcatcggcaacaagccagagtccgacatcctggtgcacaccgcctacgacgagtccaccgacgagaacgtcatgctcctgaccagcgacgccccagagtacaagccatgggccctcgtcatccaggacagcaacggggagaacaagatcaagatgctgtcgggggggagcccaaagaagaagcggaaggtg

**Figure S1**

**
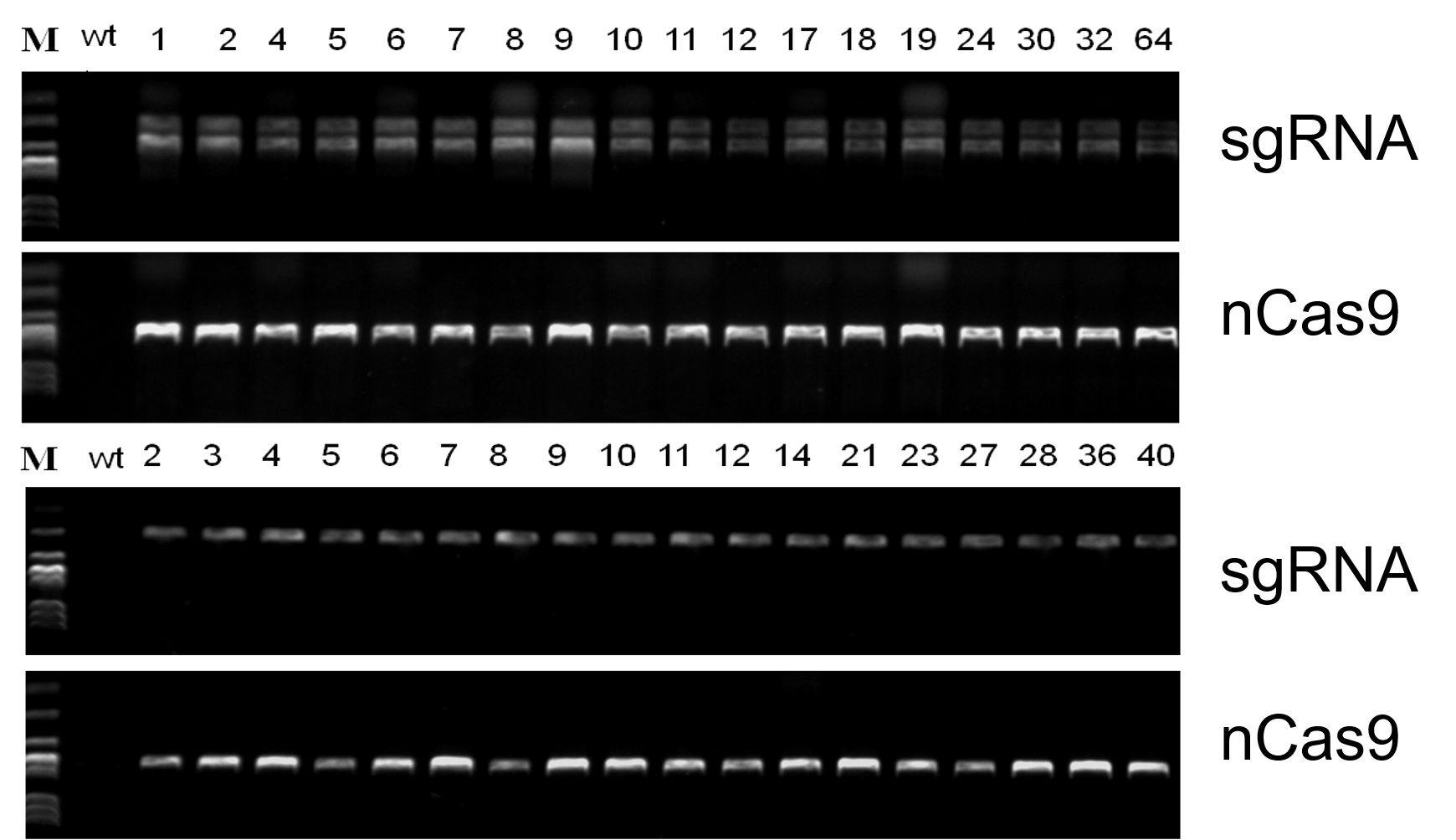
**

**Figure S1. PCR analysis for putative transgenic T0 plants with nCas9 and sgRNA specific primers.**

**Figure S2**

**
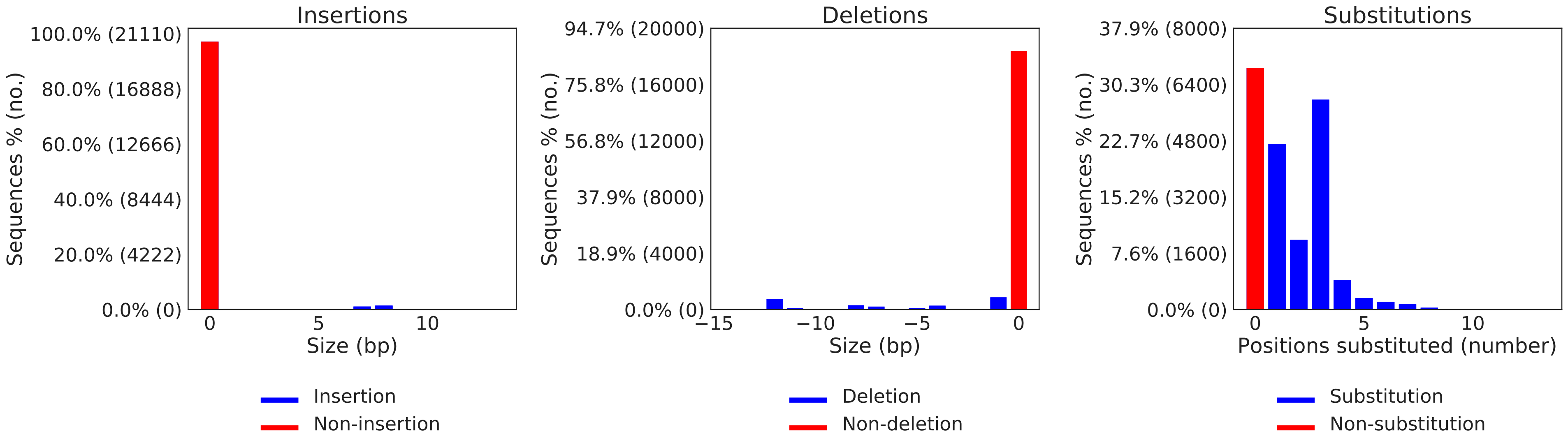
**

**Figure S2. Insertion, deletion sizes and substitutions at sgRNA2 target site in the *GhCLA*.**

**Table S1. Summary of target genes edited by GhBE3 base editor**

| **Targets** | **Chr** | **start** | **end** | **Chr** | **start** | **end** |
| --- | --- | --- | --- | --- | --- | --- |
| sgRNA1 | scaffold2473_A10 | 1406283 | 1406302 | D10 | 45350559 | 45350578 |
| sgRNA2 | scaffold2473_A10 | 1405256 | 1405275 | D10 | 45351593 | 45351612 |
| sgRNA3 | D07 | 15445062 | 15445081 | - | - | - |

**Table S2, Table S3, Table S4 ( Separate Excel file)**

**Table S5. Barcode primers for the detection of on-target editing in T0 plants** **by deep sequencing.**

|  | **Plants** | **Forward primers** | **Reverse primers** |
| --- | --- | --- | --- |
|  | CLA1 | CTGCAGGATTAAACCTCTTCCTCCGCC | TGAGCGTGCAAATCTTACAGGCAGCTTCT |
|  | CLA2 | CAGTCGGATTAAACCTCTTCCTCCGCC | TGAGCGTGCAAATCTTACAGGCAGCTTCT |
|  | CLA3 | GTCAGCGATTAAACCTCTTCCTCCGCC | TGAGCGTGCAAATCTTACAGGCAGCTTCT |
|  | CLA4 | GACGTCGATTAAACCTCTTCCTCCGCC | TGAGCGTGCAAATCTTACAGGCAGCTTCT |
|  | CLA5 | TGACGCGATTAAACCTCTTCCTCCGCC | TGAGCGTGCAAATCTTACAGGCAGCTTCT |
|  | CLA6 | ACGTCGGATTAAACCTCTTCCTCCGCC | TGAGCGTGCAAATCTTACAGGCAGCTTCT |
|  | CLA7 | AGCTGCGATTAAACCTCTTCCTCCGCC | TGAGCGTGCAAATCTTACAGGCAGCTTCT |
|  | CLA8 | CTGCAGGATTAAACCTCTTCCTCCGCC | TAGCGCTGCAAATCTTACAGGCAGCTTCT |
|  | CLA9 | CAGTCGGATTAAACCTCTTCCTCCGCC | TAGCGCTGCAAATCTTACAGGCAGCTTCT |
|  | CLA10 | GTCAGCGATTAAACCTCTTCCTCCGCC | TAGCGCTGCAAATCTTACAGGCAGCTTCT |
|  | CLA11 | GACGTCGATTAAACCTCTTCCTCCGCC | TAGCGCTGCAAATCTTACAGGCAGCTTCT |
|  | CLA12 | TGACGCGATTAAACCTCTTCCTCCGCC | TAGCGCTGCAAATCTTACAGGCAGCTTCT |
|  | CLA13 | ACGTCGGATTAAACCTCTTCCTCCGCC | TAGCGCTGCAAATCTTACAGGCAGCTTCT |
|  | CLA14 | AGCTGCGATTAAACCTCTTCCTCCGCC | TAGCGCTGCAAATCTTACAGGCAGCTTCT |
|  | CLA15 | CTGCAGGATTAAACCTCTTCCTCCGCC | CACTGCTGCAAATCTTACAGGCAGCTTCT |
|  | CLA16 | CAGTCGGATTAAACCTCTTCCTCCGCC | CACTGCTGCAAATCTTACAGGCAGCTTCT |
|  | CLA17 | GTCAGCGATTAAACCTCTTCCTCCGCC | CACTGCTGCAAATCTTACAGGCAGCTTCT |
|  | CLA18 | GACGTCGATTAAACCTCTTCCTCCGCC | CACTGCTGCAAATCTTACAGGCAGCTTCT |
|  | CLA19 | TGACGCGATTAAACCTCTTCCTCCGCC | CACTGCTGCAAATCTTACAGGCAGCTTCT |
|  | CLA20 | ACGTCGGATTAAACCTCTTCCTCCGCC | CACTGCTGCAAATCTTACAGGCAGCTTCT |
|  | CLA21 | AGCTGCGATTAAACCTCTTCCTCCGCC | CACTGCTGCAAATCTTACAGGCAGCTTCT |
| sgRNA1 | CLA22 | CTGCAGGATTAAACCTCTTCCTCCGCC | CAGTCGTGCAAATCTTACAGGCAGCTTCT |
|  | CLA23 | CAGTCGGATTAAACCTCTTCCTCCGCC | CAGTCGTGCAAATCTTACAGGCAGCTTCT |
|  | CLA24 | GTCAGCGATTAAACCTCTTCCTCCGCC | CAGTCGTGCAAATCTTACAGGCAGCTTCT |
|  | CLA25 | GACGTCGATTAAACCTCTTCCTCCGCC | CAGTCGTGCAAATCTTACAGGCAGCTTCT |
|  | CLA26 | TGACGCGATTAAACCTCTTCCTCCGCC | CAGTCGTGCAAATCTTACAGGCAGCTTCT |
|  | CLA27 | ACGTCGGATTAAACCTCTTCCTCCGCC | CAGTCGTGCAAATCTTACAGGCAGCTTCT |
|  | CLA28 | AGCTGCGATTAAACCTCTTCCTCCGCC | CAGTCGTGCAAATCTTACAGGCAGCTTCT |
|  | CLA29 | CTGCAGGATTAAACCTCTTCCTCCGCC | GTACGCTGCAAATCTTACAGGCAGCTTCT |
|  | CLA30 | CAGTCGGATTAAACCTCTTCCTCCGCC | GTACGCTGCAAATCTTACAGGCAGCTTCT |
|  | CLA31 | GTCAGCGATTAAACCTCTTCCTCCGCC | GTACGCTGCAAATCTTACAGGCAGCTTCT |
|  | CLA32 | GACGTCGATTAAACCTCTTCCTCCGCC | GTACGCTGCAAATCTTACAGGCAGCTTCT |
|  | CLA33 | TGACGCGATTAAACCTCTTCCTCCGCC | GTACGCTGCAAATCTTACAGGCAGCTTCT |
|  | CLA34 | ACGTCGGATTAAACCTCTTCCTCCGCC | GTACGCTGCAAATCTTACAGGCAGCTTCT |
|  | CLA35 | AGCTGCGATTAAACCTCTTCCTCCGCC | GTACGCTGCAAATCTTACAGGCAGCTTCT |
|  | CLA36 | CTGCAGGATTAAACCTCTTCCTCCGCC | GATCGATGCAAATCTTACAGGCAGCTTCT |
|  | CLA37 | CAGTCGGATTAAACCTCTTCCTCCGCC | GATCGATGCAAATCTTACAGGCAGCTTCT |
|  | CLA38 | GTCAGCGATTAAACCTCTTCCTCCGCC | GATCGATGCAAATCTTACAGGCAGCTTCT |
|  | CLA39 | GACGTCGATTAAACCTCTTCCTCCGCC | GATCGATGCAAATCTTACAGGCAGCTTCT |
|  | CLA40 | TGACGCGATTAAACCTCTTCCTCCGCC | GATCGATGCAAATCTTACAGGCAGCTTCT |
|  | CLA41 | ACGTCGGATTAAACCTCTTCCTCCGCC | GATCGATGCAAATCTTACAGGCAGCTTCT |
|  | CLA42 | AGCTGCGATTAAACCTCTTCCTCCGCC | GATCGATGCAAATCTTACAGGCAGCTTCT |
|  | CLA43  CLA44  CLA45 | CTGCAGGATTAAACCTCTTCCTCCGCC | ACGTCGTGCAAATCTTACAGGCAGCTTCT |
|  | **Plants** | **Forward primers** | **Reverse primers** |
|  | CLA1 | CGCTAGGCAAAGTAAAGGTATGCGAATC | CATCAGAGCCTCTCTTTTTTGTTCAAGC |
|  | CLA2 | TGCAGCGCAAAGTAAAGGTATGCGAATC | CATCAGAGCCTCTCTTTTTTGTTCAAGC |
|  | CLA3 | GCATGCGCAAAGTAAAGGTATGCGAATC | CATCAGAGCCTCTCTTTTTTGTTCAAGC |
|  | CLA4 | CAGCTGGCAAAGTAAAGGTATGCGAATC | CATCAGAGCCTCTCTTTTTTGTTCAAGC |
|  | CLA5 | GACGTCGCAAAGTAAAGGTATGCGAATC | CATCAGAGCCTCTCTTTTTTGTTCAAGC |
|  | CLA6 | GCATGCGCAAAGTAAAGGTATGCGAATC | CTAGTCAGCCTCTCTTTTTTGTTCAAGC |
|  | CLA7 | CGCTAGGCAAAGTAAAGGTATGCGAATC | CTAGTCAGCCTCTCTTTTTTGTTCAAGC |
|  | CLA8 | CAGCTGGCAAAGTAAAGGTATGCGAATC | CTAGTCAGCCTCTCTTTTTTGTTCAAGC |
|  | CLA9 | TGCAGCGCAAAGTAAAGGTATGCGAATC | CTAGTCAGCCTCTCTTTTTTGTTCAAGC |
|  | CLA10 | GACGTCGCAAAGTAAAGGTATGCGAATC | CTAGTCAGCCTCTCTTTTTTGTTCAAGC |
|  | CLA11 | GCATGCGCAAAGTAAAGGTATGCGAATC | ACTACGAGCCTCTCTTTTTTGTTCAAGC |
|  | CLA12 | CGCTAGGCAAAGTAAAGGTATGCGAATC | ACTACGAGCCTCTCTTTTTTGTTCAAGC |
|  | CLA13 | CAGCTGGCAAAGTAAAGGTATGCGAATC | ACTACGAGCCTCTCTTTTTTGTTCAAGC |
|  | CLA14 | GCATGCGCAAAGTAAAGGTATGCGAATC | AGCTAGAGCCTCTCTTTTTTGTTCAAGC |
|  | CLA15 | TGCAGCGCAAAGTAAAGGTATGCGAATC | ACTACGAGCCTCTCTTTTTTGTTCAAGC |
|  | CLA16 | CAGCTGGCAAAGTAAAGGTATGCGAATC | AGCTAGAGCCTCTCTTTTTTGTTCAAGC |
|  | CLA17 | GCATGCGCAAAGTAAAGGTATGCGAATC | TCGATGAGCCTCTCTTTTTTGTTCAAGC |
|  | CLA18 | GACGTCGCAAAGTAAAGGTATGCGAATC | ACTACGAGCCTCTCTTTTTTGTTCAAGC |
|  | CLA19 | CGCTAGGCAAAGTAAAGGTATGCGAATC | AGCTAGAGCCTCTCTTTTTTGTTCAAGC |
|  | CLA20 | CAGCTGGCAAAGTAAAGGTATGCGAATC | TCGATGAGCCTCTCTTTTTTGTTCAAGC |
|  | CLA21 | TGCAGCGCAAAGTAAAGGTATGCGAATC | AGCTAGAGCCTCTCTTTTTTGTTCAAGC |
| sgRNA2 | CLA22 | GACGTCGCAAAGTAAAGGTATGCGAATC | AGCTAGAGCCTCTCTTTTTTGTTCAAGC |
|  | CLA23 | GCATGCGCAAAGTAAAGGTATGCGAATC | TGACGTAGCCTCTCTTTTTTGTTCAAGC |
|  | CLA24 | CGCTAGGCAAAGTAAAGGTATGCGAATC | TCGATGAGCCTCTCTTTTTTGTTCAAGC |
|  | CLA25 | TGCAGCGCAAAGTAAAGGTATGCGAATC | TCGATGAGCCTCTCTTTTTTGTTCAAGC |
|  | CLA26 | GACGTCGCAAAGTAAAGGTATGCGAATC | TCGATGAGCCTCTCTTTTTTGTTCAAGC |
|  | CLA27 | CAGCTGGCAAAGTAAAGGTATGCGAATC | TGACGTAGCCTCTCTTTTTTGTTCAAGC |
|  | CLA28 | GCATGCGCAAAGTAAAGGTATGCGAATC | GTCAGCAGCCTCTCTTTTTTGTTCAAGC |
|  | CLA29 | CGCTAGGCAAAGTAAAGGTATGCGAATC | TGACGTAGCCTCTCTTTTTTGTTCAAGC |
|  | CLA30 | TGCAGCGCAAAGTAAAGGTATGCGAATC | TGACGTAGCCTCTCTTTTTTGTTCAAGC |
|  | CLA31 | CAGCTGGCAAAGTAAAGGTATGCGAATC | GTCAGCAGCCTCTCTTTTTTGTTCAAGC |
|  | CLA32 | GACGTCGCAAAGTAAAGGTATGCGAATC | TGACGTAGCCTCTCTTTTTTGTTCAAGC |
|  | CLA33 | GCATGCGCAAAGTAAAGGTATGCGAATC | GTACTGAGCCTCTCTTTTTTGTTCAAGC |
|  | CLA34 | CAGCTGGCAAAGTAAAGGTATGCGAATC | GTACTGAGCCTCTCTTTTTTGTTCAAGC |
|  | CLA35 | CGCTAGGCAAAGTAAAGGTATGCGAATC | GTCAGCAGCCTCTCTTTTTTGTTCAAGC |
|  | CLA36 | GACGTCGCAAAGTAAAGGTATGCGAATC | TGCAGCAGCCTCTCTTTTTTGTTCAAGC |
|  | CLA37 | TGCAGCGCAAAGTAAAGGTATGCGAATC | GTCAGCAGCCTCTCTTTTTTGTTCAAGC |
|  | CLA38 | GACGTCGCAAAGTAAAGGTATGCGAATC | GTCAGCAGCCTCTCTTTTTTGTTCAAGC |
|  | CLA39 | CGCTAGGCAAAGTAAAGGTATGCGAATC | GTACTGAGCCTCTCTTTTTTGTTCAAGC |
|  | CLA40 | TGCAGCGCAAAGTAAAGGTATGCGAATC | GTACTGAGCCTCTCTTTTTTGTTCAAGC |
|  | CLA41 | GACGTCGCAAAGTAAAGGTATGCGAATC | GTACTGAGCCTCTCTTTTTTGTTCAAGC |
|  | CLA42 | GCATGCGCAAAGTAAAGGTATGCGAATC | TGCAGCAGCCTCTCTTTTTTGTTCAAGC |
|  | CLA43 | CAGCTGGCAAAGTAAAGGTATGCGAATC | TGCAGCAGCCTCTCTTTTTTGTTCAAGC |
|  | **Lines** | **Forward primer** | **Reverse primer** |
|  | PEBP1 | ACGTCGTACACACAGGAAGGGAAATGGT | CATCTGAACGATCCCTTGATGACGGTA |
|  | PEBP2 | TGCAGCTACACACAGGAAGGGAAATGGT | CATCTGAACGATCCCTTGATGACGGTA |
|  | PEBP3 | GACGTCTACACACAGGAAGGGAAATGGT | CATCTGAACGATCCCTTGATGACGGTA |
|  | PEBP4 | GCATGCTACACACAGGAAGGGAAATGGT | CATCTGAACGATCCCTTGATGACGGTA |
|  | PEBP5 | CAGCTGTACACACAGGAAGGGAAATGGT | CATCTGAACGATCCCTTGATGACGGTA |
|  | PEBP6 | ACGACTTACACACAGGAAGGGAAATGGT | CATCTGAACGATCCCTTGATGACGGTA |
|  | PEBP7 | ACGTCGTACACACAGGAAGGGAAATGGT | CTAGTCAACGATCCCTTGATGACGGTA |
|  | PEBP8 | TGCAGCTACACACAGGAAGGGAAATGGT | CTAGTCAACGATCCCTTGATGACGGTA |
|  | PEBP9 | GACGTCTACACACAGGAAGGGAAATGGT | CTAGTCAACGATCCCTTGATGACGGTA |
|  | PEBP10 | GCATGCTACACACAGGAAGGGAAATGGT | CTAGTCAACGATCCCTTGATGACGGTA |
|  | PEBP11 | CAGCTGTACACACAGGAAGGGAAATGGT | CTAGTCAACGATCCCTTGATGACGGTA |
|  | PEBP12 | ACGACTTACACACAGGAAGGGAAATGGT | CTAGTCAACGATCCCTTGATGACGGTA |
|  | PEBP13 | ACGTCGTACACACAGGAAGGGAAATGGT | ACTACGAACGATCCCTTGATGACGGTA |
|  | PEBP14 | TGCAGCTACACACAGGAAGGGAAATGGT | ACTACGAACGATCCCTTGATGACGGTA |
|  | PEBP15 | GACGTCTACACACAGGAAGGGAAATGGT | ACTACGAACGATCCCTTGATGACGGTA |
|  | PEBP16 | GCATGCTACACACAGGAAGGGAAATGGT | ACTACGAACGATCCCTTGATGACGGTA |
|  | PEBP17 | CAGCTGTACACACAGGAAGGGAAATGGT | ACTACGAACGATCCCTTGATGACGGTA |
|  | PEBP18 | ACGACTTACACACAGGAAGGGAAATGGT | ACTACGAACGATCCCTTGATGACGGTA |
|  | PEBP19 | ACGTCGTACACACAGGAAGGGAAATGGT | AGCTAGAACGATCCCTTGATGACGGTA |
|  | PEBP20 | TGCAGCTACACACAGGAAGGGAAATGGT | AGCTAGAACGATCCCTTGATGACGGTA |
|  | PEBP21 | GACGTCTACACACAGGAAGGGAAATGGT | AGCTAGAACGATCCCTTGATGACGGTA |
| sgRNA3 | PEBP22 | GCATGCTACACACAGGAAGGGAAATGGT | AGCTAGAACGATCCCTTGATGACGGTA |
|  | PEBP23 | ACGACTTACACACAGGAAGGGAAATGGT | AGCTAGAACGATCCCTTGATGACGGTA |
|  | PEBP24 | ACGTCGTACACACAGGAAGGGAAATGGT | TCGATGAACGATCCCTTGATGACGGTA |
|  | PEBP25 | TGCAGCTACACACAGGAAGGGAAATGGT | TCGATGAACGATCCCTTGATGACGGTA |
|  | PEBP26 | GACGTCTACACACAGGAAGGGAAATGGT | TCGATGAACGATCCCTTGATGACGGTA |
|  | PEBP27 | GCATGCTACACACAGGAAGGGAAATGGT | TCGATGAACGATCCCTTGATGACGGTA |
|  | PEBP28 | CAGCTGTACACACAGGAAGGGAAATGGT | TCGATGAACGATCCCTTGATGACGGTA |
|  | PEBP29 | ACGACTTACACACAGGAAGGGAAATGGT | TCGATGAACGATCCCTTGATGACGGTA |
|  | PEBP30 | ACGTCGTACACACAGGAAGGGAAATGGT | GTCAGCAACGATCCCTTGATGACGGTA |
|  | PEBP31 | TGCAGCTACACACAGGAAGGGAAATGGT | GTCAGCAACGATCCCTTGATGACGGTA |
|  | PEBP32 | GACGTCTACACACAGGAAGGGAAATGGT | GTCAGCAACGATCCCTTGATGACGGTA |
|  | PEBP33 | GCATGCTACACACAGGAAGGGAAATGGT | GTCAGCAACGATCCCTTGATGACGGTA |
|  | PEBP34 | CAGCTGTACACACAGGAAGGGAAATGGT | GTCAGCAACGATCCCTTGATGACGGTA |
|  | PEBP35 | ACGACTTACACACAGGAAGGGAAATGGT | GTCAGCAACGATCCCTTGATGACGGTA |
|  | PEBP36 | ACGTCGTACACACAGGAAGGGAAATGGT | GTACTGAACGATCCCTTGATGACGGTA |
|  | PEBP37 | GACGTCTACACACAGGAAGGGAAATGGT | GTACTGAACGATCCCTTGATGACGGTA |
|  | PEBP38 | GCATGCTACACACAGGAAGGGAAATGGT | GTACTGAACGATCCCTTGATGACGGTA |
|  | PEBP39 | CAGCTGTACACACAGGAAGGGAAATGGT | GTACTGAACGATCCCTTGATGACGGTA |
|  | PEBP40 | ACGACTTACACACAGGAAGGGAAATGGT | GTACTGAACGATCCCTTGATGACGGTA |

**Note:** the six base that highlighted by red was barcode, a pair of different barcode primer corresponds to an independent plant.

**Table S6. Barcode primers for the detection of off-target in T0 plants by deep** sequencing

| **Gene** | **Plants** | **Forward primers** | **Reverse primers** |
| --- | --- | --- | --- |
| **Ghir_**  **A13** | CLA2 | CGCTAGCATCTAATGCCCTTACTGTGGAAT | CATCAGTGGGTGAATAATCTAAACACGGAT |
|  | CLA5 | TGCAGCCATCTAATGCCCTTACTGTGGAAT | CATCAGTGGGTGAATAATCTAAACACGGAT |
|  | CLA7 | GACGTCCATCTAATGCCCTTACTGTGGAAT | CATCAGTGGGTGAATAATCTAAACACGGAT |
|  | CLA10 | CGCTAGCATCTAATGCCCTTACTGTGGAAT | CTAGTCTGGGTGAATAATCTAAACACGGAT |
|  | CLA15 | TGCAGCCATCTAATGCCCTTACTGTGGAAT | CTAGTCTGGGTGAATAATCTAAACACGGAT |
|  | CLA18 | GACGTCCATCTAATGCCCTTACTGTGGAAT | CTAGTCTGGGTGAATAATCTAAACACGGAT |
|  | CLA25 | CGCTAGCATCTAATGCCCTTACTGTGGAAT | ACTACGTGGGTGAATAATCTAAACACGGAT |
|  | CLA26 | TGCAGCCATCTAATGCCCTTACTGTGGAAT | ACTACGTGGGTGAATAATCTAAACACGGAT |
|  | CLA27 | GACGTCCATCTAATGCCCTTACTGTGGAAT | ACTACGTGGGTGAATAATCTAAACACGGAT |
|  | CLA32 | CGCTAGCATCTAATGCCCTTACTGTGGAAT | CATCAGTGGGTGAATAATCTAAACACGGAT |
| **Ghir_**  **D04** | CLA2 | GCATGCGAGATCGACATGGCTCAAAAC | CACTGCCCAATCAACACCCTTCAATACTT |
|  | CLA5 | CAGCTGGAGATCGACATGGCTCAAAAC | CACTGCCCAATCAACACCCTTCAATACTT |
|  | CLA7 | ACGACTGAGATCGACATGGCTCAAAAC | CACTGCCCAATCAACACCCTTCAATACTT |
|  | CLA10 | GCATGCGAGATCGACATGGCTCAAAAC | TGACGTCCAATCAACACCCTTCAATACTT |
|  | CLA15 | CAGCTGGAGATCGACATGGCTCAAAAC | TGACGTCCAATCAACACCCTTCAATACTT |
|  | CLA18 | ACGACTGAGATCGACATGGCTCAAAAC | TGACGTCCAATCAACACCCTTCAATACTT |
|  | CLA25 | GCATGCGAGATCGACATGGCTCAAAAC | GTCAGCCCAATCAACACCCTTCAATACTT |
|  | CLA26 | CAGCTGGAGATCGACATGGCTCAAAAC | GTCAGCCCAATCAACACCCTTCAATACTT |
|  | CLA27 | ACGACTGAGATCGACATGGCTCAAAAC | GTCAGCCCAATCAACACCCTTCAATACTT |
|  | CLA32 | GCATGCGAGATCGACATGGCTCAAAAC | GTACTGCCAATCAACACCCTTCAATACTT |
| **Ghir_A10** | CLA2 | ACGTCGTCCACTATGGCTTCCAAAGATGA | TGCAGCCCAGTGCAGTTGTGCGAAAT |
|  | CLA5 | CGCTAGTCCACTATGGCTTCCAAAGATGA | TGCAGCCCAGTGCAGTTGTGCGAAAT |
|  | CLA7 | TGCAGCTCCACTATGGCTTCCAAAGATGA | TGCAGCCCAGTGCAGTTGTGCGAAAT |
|  | CLA10 | ACGTCGTCCACTATGGCTTCCAAAGATGA | CATCAGCCAGTGCAGTTGTGCGAAAT |
|  | CLA15 | CGCTAGTCCACTATGGCTTCCAAAGATGA | CATCAGCCAGTGCAGTTGTGCGAAAT |
|  | CLA18 | TGCAGCTCCACTATGGCTTCCAAAGATGA | CATCAGCCAGTGCAGTTGTGCGAAAT |
|  | CLA25 | ACGTCGTCCACTATGGCTTCCAAAGATGA | CTAGTCCCAGTGCAGTTGTGCGAAAT |
|  | CLA26 | CGCTAGTCCACTATGGCTTCCAAAGATGA | CTAGTCCCAGTGCAGTTGTGCGAAAT |
|  | CLA27 | TGCAGCTCCACTATGGCTTCCAAAGATGA | CTAGTCCCAGTGCAGTTGTGCGAAAT |
|  | CLA32 | ACGTCGTCCACTATGGCTTCCAAAGATGA | ACTACGCCAGTGCAGTTGTGCGAAAT |
| **Ghir_D03** | CLA2 | GACGTCCATCACGGGACAGTGTTCAGT | ACTACGATCTTACAACATAGGCAAGGGAA |
|  | CLA5 | GCATGCCATCACGGGACAGTGTTCAGT | ACTACGATCTTACAACATAGGCAAGGGAA |
|  | CLA7 | CAGCTGCATCACGGGACAGTGTTCAGT | ACTACGATCTTACAACATAGGCAAGGGAA |
|  | CLA10 | GACGTCCATCACGGGACAGTGTTCAGT | AGCTAGATCTTACAACATAGGCAAGGGAA |
|  | CLA15 | GCATGCCATCACGGGACAGTGTTCAGT | AGCTAGATCTTACAACATAGGCAAGGGAA |
|  | CLA18 | CAGCTGCATCACGGGACAGTGTTCAGT | AGCTAGATCTTACAACATAGGCAAGGGAA |
|  | CLA25 | GACGTCCATCACGGGACAGTGTTCAGT | TCGATGATCTTACAACATAGGCAAGGGAA |
|  | CLA26 | GCATGCCATCACGGGACAGTGTTCAGT | TCGATGATCTTACAACATAGGCAAGGGAA |
|  | CLA27 | CAGCTGCATCACGGGACAGTGTTCAGT | TCGATGATCTTACAACATAGGCAAGGGAA |
|  | CLA32 | GACGTCCATCACGGGACAGTGTTCAGT | ACGTCGATCTTACAACATAGGCAAGGGAA |

**Note：**The six bases that highlighted by red font are barcodes and a pair of barcode primers corresponds to an independent plant.

**Table S7. Primers used for vectors construction, PCR test and target region amplification.**

| **primers** | **Sequence (5'-3')** |
| --- | --- |
| BE3/F | AAAAAGCAGGCTTCGATGCCAAAGAAGAAGAGGAAG |
| BE3/R | GAAAGCTGGGTCTAGACCGATGATACGAACGAAAG |
| nCas9/F | CTGGTTGTGGCGAAGGTTGA |
| nCas9/R | TTCTTGGTTGCTGCGGGTCT |
| gRNA/F | CGTGGTAGCATACTTCAGGGAT |
| gRNA/R | TTCAAGTTGATAACGGACTAGCC |
| CLA/F | TGCTGACAAGTATCACGGTAA |
| CLA/R | ATAGATGTGATTGACTTATGTGGA |
| PEBP/F | TACACACAGGAAGGGAAATGGT |
| PEBP/R | AACGATCCCTTGATGACGGTA |
| VnCas9/F | AAAGCAGGCTTCGATGC |
| VnCas9/R  CLA1/F  CLA1/R | GCTGGGTCTAGACCGATGATA  AAGTAAAGGTATGCGAATCCG  GGGTTGTTTTTAACTTTTCTCG |

**Table S8. The nine most likely off-target sites of each sgRNA target sites in cotton genome.**

**Note**: Mismatch bases of sgRNA between the WT and off-target sequences are marked with lowercase letters，and all C bases in editing window of off-target are highlighted in red font.

**Table S9. The summary of variation calling between wild-type and GhBE3 edited plants by the samtools and GATK.**

sgRNA1

| Sample | Samtools | GATK | Comm | Concordance |
| --- | --- | --- | --- | --- |
| WT | 3,123,071 | 3,878,241 | 2,937,036 | 976,280 |
| N17 | 3,331,752 | 4,495,667 | 3,275,317 | 1,124,776 |
| N64 | 3,320,713 | 4,488,673 | 3,263,633 | 1,138,453 |

sgRNA2

| Sample | Samtools | GATK | Comm | Concordance |
| --- | --- | --- | --- | --- |
| WT | 286,126 | 282,118 | 224,767 | 134,097 |
| N17 | 319,157 | 309,905 | 240,995 | 139,787 |
| N64 | 320,372 | 308,635 | 240,039 | 140,733 |
